# The Beauty and the Beast: Bariatric Surgery and a Case of Oxalate Nephropathy

**DOI:** 10.7759/cureus.36801

**Published:** 2023-03-28

**Authors:** Renata Carvalho, Joana Medeiros, Bárbara Ribeiro, José Mário Bastos, Raquel Vaz

**Affiliations:** 1 Nephrology Department, Hospital de Braga, Braga, PRT

**Keywords:** roux-en-y gastric bypass surgery, kidney injury, rygb, bariatric surgery, obesity, oxalate nephropathy

## Abstract

Oxalate nephropathy is a rare cause of kidney failure. Roux-en-Y gastric bypass surgery is a technique used for surgical treatment of obesity as well as for the treatment of gastric carcinoma. We report the case of a 46-year-old male who was admitted to the nephrology department due to kidney dysfunction eight months after bariatric surgery.

## Introduction

Oxalate nephropathy is a rare cause of kidney failure, which is characterized by the presence of tubular crystalline deposits of calcium oxalate. Oxalate nephropathy can be primary (hereditary), causing a rare multisystemic disease, or secondary, as a result of excessive ingestion of dietary sources of oxalate, after ethylene glycol intoxication, due to ingestion of high amounts of vitamin C, or due to enteric hyperoxaluria [1,2]. Enteric hyperoxaluria occurs due to the malabsorption of fat or bile acids that bind to calcium ions. Normally, calcium combines with oxalate and forms calcium oxalate complexes that are excreted in the gastrointestinal tract. In enteric hyperoxaluria, calcium is linked to fat and bile acids and oxalate is free to be absorbed systemically. Roux-en-Y gastric bypass (RYGB) surgery is considered to be a restrictive as well as a malabsorptive technique used in bariatric and gastric carcinoma treatment.

## Case presentation

A 46-year-old male was admitted to the nephrology department with serum creatinine (sCr) of 13.4 mg/dL (normal range 0.8-1.2 mg/dL), anemia, with hemoglobin (Hb) of 7.6 g/dL (normal range 13.5-17.0 g/dL), normal serum calcium 8.3 mg/dl (normal range 8.3-10.6 mg/dL) and phosphorus 5.0 mg/dl (<5.1 mg/dL). He mentioned cramps that improved after magnesium supplementation and asthenia in the previous month. His stool had become looser after bariatric surgery, but he denied diarrhea or steatorrhea. He denied nausea, vomits, fever, urinary symptoms, rash, purpura, arthralgias, mouth sores, eye inflammation, and a history of renal stones. His past medical history included hypertension for 10 years, obesity, obstructive sleep apnea, and an isthmectomy due to follicular adenoma eight years prior. He had no chronic medication and no nephrotoxic agents were identified. RYGB was eight months before admission and in pre-operative laboratory assessment he had sCr of 0.94 mg/dL and Hb of 14.5 g/dL. After surgery, the patient lost about 50 kilograms, 100% of his excess body weight.

On physical examination, he was pale, his arterial pressure was 150/90 mmHg, with other vital signs stable. He had a few rales in pulmonary bases but no peripheral edema. The remaining clinical examination was unremarkable. Immunological study was negative, with antinuclear antibodies (ANA), antineutrophil cytoplasmic antibodies (ANCA), anti-double stranded (ds)-DNA, anti-glomerular basement membrane antibodies, serum complement, serum immunoglobulins, and serum electrophoresis with normal results. Viral serology for human immunodeficiency virus, hepatitis C virus, and hepatitis B virus were negative. Urine sediment had no cells and proteinuria was 800 mg/day. Renal ultrasound showed kidneys with normal size and increased parenchyma echogenicity, without hydronephrosis, cysts, or stones.

**Figure 1:**
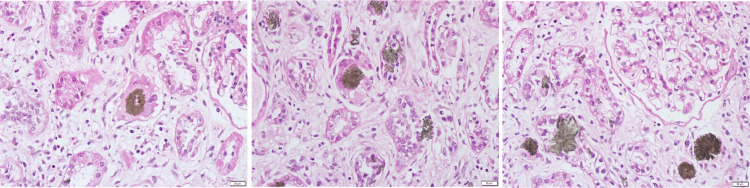
Calcium oxalate crystals deposition in tubules and interstitium with giant multinucleated cells reaction (hematoxylin and eosin, x400).

Renal biopsy was performed and showed 16 glomeruli of which seven were globally sclerosed. The remaining glomeruli had no changes. Tubular interstitial compartment showed multinucleated giant cells reaction with calcium oxalate crystals deposited in tubules and interstitium (Figure 1); the deposits were birefringent under polarized light. There was interstitial fibrosis in 30% of the parenchyma. Immunofluorescence study showed diffuse fibrinogen positivity, with negative fixation for immunoglobulins, free light chains and complement proteins.

A 24-hour urine collection was performed and showed calcium slightly reduced (80 mg), while citrate and oxalate levels were normal (161 mg and 20 mg, respectively). The patient was diagnosed with oxalate nephropathy secondary to enteric hyperoxaluria. Renal impairment persisted despite the adoption of a low oxalate diet and the start of oral calcium supplements with meals. The patient was started on hemodialysis without recovery of kidney function.

## Discussion

The incidence of obesity is increasing and bariatric surgeries are commonly practiced with a great reduction of weight. The benefits of bariatric surgery are diverse and include remission of diabetes and dyslipidemia and improvement in hypertension control and chronic kidney disease progression [3-5].

Jejunoileal bypass was one of the first bariatric surgeries, but it was abandoned due to high rates of complications including diarrhea, electrolyte imbalances, nephrolithiasis, and oxalate nephropathy [6]. RYGB is now one of the most used techniques in bariatric surgery. The first described case of oxalate nephropathy after RYGB was in 2005 and few cases have been described in the literature [7]. Nasr et al. described 11 patients with oxalate nephropathy after RYGB of which seven patients also suffered from diabetic glomerulosclerosis [6]. This suggests previous kidney disease is a risk factor for the formation of calcium oxalate crystals and tubulointerstitial injury. However, our patient was not diabetic and his kidney biopsy did not show diabetic changes.

In the case series described previously, kidney failure was observed four to 96 months after surgery, with described progression to end-stage renal disease (ESRD) within three months in 72.7% [6]. In our case, the patient presented with severe kidney dysfunction and anemia eight months after RYGB surgery. Despite that, he had few symptoms, which could let us think that the progression was slow allowing for adaptation.

Few studies show an increase in urinary oxalate excretion after RYGB [8]. In our case, urinary oxalate was normal. Urine collection after starting a low oxalate diet could have influenced the results. 

Treatment of enteric oxalate nephropathy includes a reduction in ingestion of oxalate-rich food and fat and, when possible, hydration should be optimized to decrease urine supersaturation. Calcium supplementation can increase intestinal elimination of calcium oxalate complexes. Cholestyramine, a bile acid-binding resin, is a therapeutic option to reduce bile acids and promote calcium-to-oxalate binding [1,9]. The prognosis of oxalate nephropathy is poor, with progression to ESRD in the majority of cases [1,6]. Oxalate nephropathy related to bariatric surgery is difficult to treat [10]. There is one case described of reversal of RYGB with improved kidney function [10]. Unlike our case, kidney dysfunction was less severe, with only mild interstitial inflammation and no glomerular damage.

## Conclusions

Oxalate nephropathy is a rare and underdiagnosed complication of RYGB with fast progression to ESRD. Patients submitted to RYGB should have regular follow-up of kidney function. The diagnosis should be suspected in patients with acute or chronic kidney injury after bariatric surgery or other intestinal surgeries that modify absorption. Early implementation of dietary changes and calcium supplementation can prevent the development of chronic kidney damage. When medical measures fail to reduce hyperoxaluria, bypass reversal may be necessary.

## References

[1] Verdelho M, Mendes M, Ribeiro F, Sousa Viana H, Carvalho F, Nolasco F (2016). Oxalate nephropathy following Roux-en-Y gastric bypass surgery - mini-review. Port J Nephrol Hypert.

[2] Marques S, Santos S, Fremin K, Fogo AB (2017). A case of oxalate nephropathy: when a single cause is not crystal clear. Am J Kidney Dis.

[3] Silva CF, Cohen L, Sarmento LD (2016). Effects of long-term Roux-en-Y gastric bypass on body weight and clinical metabolic comorbidities in bariatric surgery service of a university hospital. Arq Bras Cir Dig.

[4] Lee Y, Anvari S, Chu MM (2022). Improvement of kidney function in patients with chronic kidney disease and severe obesity after bariatric surgery: a systematic review and meta-analysis. Nephrology (Carlton).

[5] Nagaraju SP, Gupta A, McCormick B (2013). Oxalate nephropathy: an important cause of renal failure after bariatric surgery. Indian J Nephrol.

[6] Nasr SH, D'Agati VD, Said SM, Stokes MB, Largoza MV, Radhakrishnan J, Markowitz GS (2008). Oxalate nephropathy complicating Roux-en-Y gastric bypass: an underrecognized cause of irreversible renal failure. Clin J Am Soc Nephrol.

[7] Nelson WK, Houghton SG, Milliner DS, Lieske JC, Sarr MG (2005). Enteric hyperoxaluria, nephrolithiasis, and oxalate nephropathy: potentially serious and unappreciated complications of Roux-en-Y gastric bypass. Surg Obes Relat Dis.

[8] Duffey BG, Pedro RN, Makhlouf A (2008). Roux-en-Y gastric bypass is associated with early increased risk factors for development of calcium oxalate nephrolithiasis. J Am Coll Surg.

[9] Rosenstock JL, Joab TM, DeVita MV, Yang Y, Sharma PD, Bijol V (2022). Oxalate nephropathy: a review. Clin Kidney J.

[10] Agrawal V, Wilfong JB, Rich CE, Gibson PC (2016). Reversal of gastric bypass resolves hyperoxaluria and improves oxalate nephropathy secondary to Roux-en-Y gastric bypass. Case Rep Nephrol Dial.

